# Therapeutic strategy of third-generation autologous chondrocyte implantation for osteoarthritis

**DOI:** 10.3109/03009734.2011.552812

**Published:** 2011-04-12

**Authors:** Tomoya Kuroda, Tomoyuki Matsumoto, Yutaka Mifune, Tomoaki Fukui, Seiji Kubo, Takehiko Matsushita, Takayuki Asahara, Masahiro Kurosaka, Ryosuke Kuroda

**Affiliations:** ^1^Kobe University Graduate School of Medicine/Department of Orthopaedic Surgery, Kobe, Japan; ^2^Stem Cell Translational Research, Kobe Institute of Biomedical Research and Innovation, Kobe, Japan; ^3^Department of Regenerative Medicine Science, Tokai University School of Medicine, Tokai, Japan

**Keywords:** Chondrocyte implantation, geriatrics, knee osteoarthritis, orthopedics

## Abstract

**Background:**

Autologous chondrocyte implantation (ACI) is considered a promising choice for the treatment of cartilage defects. However, the application of ACI to osteoarthritic patients is, in general, contraindicated. The purpose of this study is to evaluate the efficiency of three-dimensionallystructured ACI (3D-ACI; CaReS) in a rat model of knee osteoarthritis (OA).

**Methods:**

OA-like degenerative changes in the articular cartilage were created by transecting the anterior cruciate ligament (ACLT) in athymic nude rats. Two weeks later, CaReS was transplanted at the cartilage injury sites created by micro-drilling in the patella groove (Chondrocyte-implanted (CI) group: CaReS collagen with human chondrocytes; Collagen group: CaReS collagen without cells; and Sham group: sham operation; *n* = 15/group).

**Results:**

Reverse Transcription Polymerase Chain Reaction (RT-PCR) analysis demonstrated the expression of human-specific type 2 collagen and Sry-type high-mobility-group box 9 (SOX9) in the CI group—not in the other groups—throughout the study period. Double immunohistochemistry for human-specific type 2 collagen and human leukocyte antigen-abacavir (HLA-ABC) at week 4 showed positive staining in the CI group only. Macroscopic assessment showed better repair at the cartilage defect sites in the CI group, compared to the other groups. Histological assessment with toluidine blue staining showed that the thickness of the articular cartilage and semi-quantitative histological scores were higher in the CI group than in the other groups up to week 20.

**Conclusions:**

We demonstrate, for the first time, that 3D-ACI is effective in repairing cartilage defects in a rat model of ACLT-induced OA.

## Introduction

Knee osteoarthritis (OA), a clinical syndrome with low-grade inflammation caused by abnormal wear of the articular cartilage, is associated with pain, destruction of joints, and/or a decrease in the synovial fluid that lubricates the joints. It is estimated that the number of patients with knee OA is more than 21 million in the US, and symptomatic OA of the knee occurs in 6% of adults aged 30 or older and 13% of people aged 60 or over (1). However, it is also well known that because of the limited capacity of the cartilage for repair, abnormal wear of the articular cartilage is a major clinical problem when the cartilage becomes damaged (2).

Recently, various therapeutic strategies including bone-marrow stimulation and transplantation of osteochondral autografts or allografts have been developed to restore articular cartilage so as to achieve a permanent repair (3). Among them, autologous chondrocyte implantation (ACI) is a promising choice for cartilage repair. The classical ACI (first generation) was originally described in 1994 (4) and approved by the US Food and Drug Administration in 1997 (5). This first-generation ACI provided significant and long-term benefits for patients in terms of diminished pain and improved function (6). In order to overcome the hypertrophy or ossification of the patched periosteum and the complex operative technique associated with first-generation ACI, a second-generation ACI was developed, in which bioengineered bilayer collagen or synthetic membranes were used in order to avoid spill-over and asymmetric distribution of chondrocytes following implantation. In some products, a periosteal flap is not needed.

Recently, further technological advances have led to a third-generation ACI, where chondrocytes are embedded into three-dimensionally constructed scaffolds (i.e. 3D environment) for cell growth. These ‘all-in-one’ grafts do not need a periosteal cover or fixing stitches and can be trimmed to exactly fit into the cartilage defect with fibrin glue (7). Indeed, several clinical studies of 3D-ACI for cartilage injury have been reported (2,4,8–10). Despite the advantages of this new technique in surgical simplicity, shorter operating time, and the possibility of performing the surgery via a mini-arthrotomy or arthroscopy, the product has contraindication for use in patients suffering from OA. However, there are several studies in which favorable clinical outcomes have been reported (7,11). Therefore, the purpose of this study is to evaluate the efficiency of 3D-ACI using a rat model of knee osteoarthritis produced by transection of the anterior cruciate ligament (ACL).

## Materials and methods

### OA animal model

The institutional animal care and committees of RIKEN Center for Developmental Biology approved all animal procedures. Female athymic nude rats (F344/N Jcl rnu/rnu, Japan clea) aged 8–10 weeks, weighing an average of 200 g, underwent ACL transection (ACLT) as previously reported (12). Animals were briefly anesthetized by an intraperitoneal injection of ketamine hydrochloride (60 mg/kg) and with ether. After shaving of the knee joint, a parapatellar incision was made, followed by lateral displacement of the patella, thereby enabling access to the joint space. The ACL was then easily visible and surgically excised. The increase in anterior displacement of the tibia in relation to the femur was used to ensure that the ligament was transected. The surgical incision was then closed. At the end of the 2-week post-surgical period, in order to confirm the progression to OA, the knee joints were opened in a parapatellar approach, and the entire knee was examined macroscopically and photographed. Then the distal femurs were removed and histologically assessed as described later.

### Preparation of chondrocytes and collagen matrix

In this study, to meet the demand supposing a clinical use, we used human chondrocytes. Human chondrocytes isolated from a healthy volunteer (28 years old, African-American) were purchased from Lonza (Basel, Switzerland). To expand these cells, 1 × 10^5^ cells were plated in 6-well dishes with standard medium (alpha-MEM; GibcoBRL, Invitrogen Japan K.K., Tokyo, Japan) containing 10% fetal bovine serum (Vitromex, San Antonio, TX, USA), 2 mM L-glutamine (GibcoBRL), 100 units/mL penicillin (GibcoBRL), and 100 μg/mL streptomycin (GibcoBRL) and incubated at 37°C with 5% CO_2_ in ambient air. The medium was replaced every 3–4 days. After culture for 14 days, the cells were harvested with 0.25% trypsin/1mM EDTA solution (GibcoBRL) and counted. CaReS (Arthro Kinetics, Krems am de Donau, Austria) is a 3D mechanically stable transplantation system based on patient-specific autologous cartilage cells. These counted cells were expanded *in vitro* according to CaReS manufacturer's protocol. In brief, they were expanded with standard medium (see above) and subsequently 2 × 10^7^ cells were mixed with 50 mL collagen MS solution, a gel neutralization solution (Arthro Kinetics, Krems am de Donau, Austria). An appropriate volume of the above solution was pipetted into microtiter plates and incubated for 20 minutes. Culture was performed at 37°C, 5% CO_2_, and 95% Relative Humidity (RH) for 10–14 days. Culture medium was removed from the gel every 3–4 days and replaced by fresh and preheated (37°C) medium. Preparation of collagen matrix without cells was conducted in the same manner as those with cells, except that no cells were seeded.

### Cell implantation and study groups

A parapatellar incision was made, and the knee joint was exposed via lateral dislocation of the patella. To make an appropriate space to mount the chondrocyte-loaded collagen implant in the trochlear groove of each femur, a 1.5-mm diameter micro-drill was used. Animals in the chondrocyte-implanted (CI) group were treated with several μm of the chondrocyte-loaded collagen gel (normal articular cartilage thickness is 0.072 ± 0.013 mm (13)) and fixed with fibrin glue (Kaketsuken, Kumamoto, Japan) to keep the implant stable. Animals in the collagen group were treated with fibrin glue-fixed collagen implants without cells. Animals in the sham group were treated with fibrin glue only (*n* = 15 each). After surgery, the rats were allowed to move freely within their cages. No animal was observed to have an abnormal gait or impaired locomotion.

### RT-PCR analysis of RNA isolated from transplantationsites

To examine human cell-derived chondrogenesis, mRNA expression for chondrogenic markers (human-specific type 2collagen (COL2) and SOX9) was assessed by RT-PCR analysis. At week 4, total RNA was obtained from the transplantation sites using Tri-zol (Life Technologies, Gaithersburg, MD, USA) according to the manufacturer's instructions. First-strand cDNA was synthesized using the RNA LA PCRKit Ver 1.1 (Takara Bio. Inc., Otsu, Japan), amplified by Taq DNA polymerase (Advantage-GC cDNA PCR Kit, Clontech and AmpliTaq Gold DNA polymerase, Applied Biosystems). PCR was performed using a PCR thermocycler (MJ research PTC-225). And hCOL2, hSOX9, and rGAPDH were amplified by Taq DNA polymerase (Advantage-GC cDNA PCR Kit, Clontech) at the following conditions: 35 cycles of 30-second initial denaturation at 94°C, annealing at 56°C for 1 minute, and 30 seconds of extension at 72°C according to the manufacturer's instructions. Subsequently, PCR products were visualized in 1.5% ethidium bromide stained agarose gels. Human chondrocytes (Lonza) were used for positive control for human-specific chondrocyte-related genes.

### Primers

To avoid interspecies cross-reactivity of the primer pairs between human and rat genes, we designed the following human-specific primers using Oligo software (Takara Bio. Inc.). None of the primer pairs showed any cross-reactivity to rat genes (data not shown).

- hCOL2 primer sequence: sense AAG CAA GTA GCG CCA ATC T; antisense GGA AGT AGG GTG CCA TAA CAC
- hSOX9 primer sequence: sense ATG TTT GGC CTG AAG CAG AGA; antisense GGC GGT ACA GGT CGA GCA TAT A
- rGAPDH primer sequence: sense CTG ATG CCC CCA TGT TCG TC; antisense CAC CCT GTT GCT GTA GCC AAA TTC G

### Tissue harvesting

Rats were euthanized with an overdose of ketamine and xylazine. Bilateral femurs were harvested and quickly embedded in optimal cutting temperature (OCT) compound (Miles Scientific, Elkhardt, IN, USA), snap-frozen in liquid nitrogen, and stored at −80°C for histochemical and immunohistochemical staining as described previously (14). Rat femurs in OCT blocks were sectioned, and 6-μm serial sections were mounted on silane-coated glass slides and air dried for 1 hour before being fixed with 4.0% paraformaldehyde at 4°C for 5 minutes and stained immediately.

### Immunohistochemical staining

To detect the chondral restoration by transplanted human cells in the rat tissue at week 4, immunohistochemistry was performed with the following human-specific antibodies; human leukocyte antigen (HLA)-ABC (Becton-Dickinson (BD) Pharmingen, Franklin Lakes, NJ, USA) to detect various lineages of human cells and hCOL2 (BD Pharmingen) to detect human cell-derived chondrocytes. Staining specificity for human cells without cross-reaction to rat cells was confirmed by histochemical staining for HLA-ABC and hCOL2 using rat and human chondrocytes (Lonza) (data not shown). The secondary antibodies for each immunostaining were as follows: Alexa Fluor 594-conjugated goat anti-mouse IgG_1_ (Molecular Probes, Invitrogen Japan K.K., Tokyo, Japan) for HLA-ABC staining and Alexa Fluor 488-conjugated goat anti-mouse IgG_2a_ (Molecular Probes) for hCOL2. DAPI solution was applied for 5 minutes for nuclear staining. Double immunohistochemistry with HLA-ABC and hCOL2 was performed to detect human chondrocytes in the articular cartilage.

### Macroscopic and histological assessment of cartilage regeneration

To confirm the sequential recovery from OA-like arthritis, we performed macroscopic and histological evaluation from 0 to 20 weeks after operation. The rats were euthanized at 4, 8, and 20 weeks after the operation. The entire knee was dissected, examined macroscopically, and photographed.

The distal parts of the femur were then fixed as described above. Each specimen was sectioned (5 μm thick) sagittally and perpendicularly to the defect. The sections were stained with toluidine blue. These sections were obtained from the center of the defect, and as many as ten such sections were prepared from each knee. The sections from each animal were examined and scored independently by three blinded observers. We evaluated chondral repair semi-quantitatively using a grading and scoring system, which is a modification of that described by Prizker et al. (15). The scale is composed of six histological gradings (0: surface intact, cartilage intact; 1: surface intact; 2: surface discontinuity; 3: vertical fissures; 4: erosion; 5: denudation; 6: deformation) and four histological stages (0: no OA activity; 1: <10% involvement (surface, area, volume); 2: 10%–25%; 3: 25%–50%; 4: >50%) assigning a total score (score = grade × stage) ranging from 1 point (normal articular cartilage) to 24 points (no repair).

### Statistical analysis

The results were statistically analyzed using a software package (Microsoft Office Excel, Microsoft Corporation, Washington, USA). All values were expressed as means ± SE. Multiple comparisons among groups were made using a one-way analysis of variance (ANOVA). *Post-hoc* analysis was performed by Fisher's PLSD test. A probability value < 0.05 was considered to denote statistical significance.

## Results

### Confirmation of ACLT model of rat osteoarthritis

**Figure 1.**
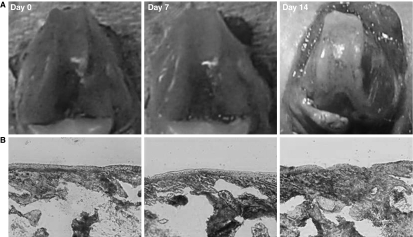
Rat osteoarthritis (OA) model with anterior cruciate ligament transection. A: Macroscopic appearance of the anterior cruciate ligament transection (ACLT) model of rat OA. At 2 weeks post-surgery, the joints have obvious articular surface damage and osteophyte formation around the margin of the medial femoral condyles and the patella groove. B: Toluidine blue staining showing the thickness of gradual degeneration of the patellar groove (Original magnification ×20).

At 2 weeks after the ACLT operation, irregular articular surface and osteophyte formation around the margin of the bilateral femoral condyle and the patella groove were confirmed (Figure 1A). Toluidine blue staining showed gradual reduction of the thickness of the articular cartilage layer in the patella groove (Figure 1B).

### Chondrogenesis by transplanted human cells

**Figure 2.**
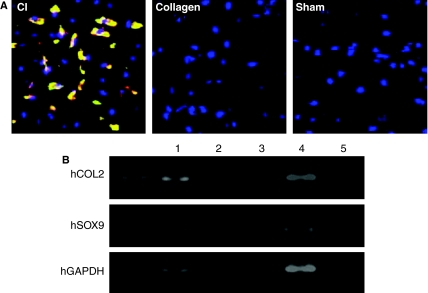
Cartilage regeneration by transplanted human cells. A: Representative double immunostaining for hCOL2 (green) and HLA-ABC (red) using tissue sample of the regenerated articular cartilage at week 4 (original magnification ×200).Human-derived chondrocytes were identified as double-stained cells with hCOL2 and HLA-ABC at the chondral repair site in the CI group (yellow area), but not in the other groups. B: RT-PCR analysis for human-specific chondrogenic markers of tissue RNA isolated from the chondral repair sites. Expression of hCOL2 and hSOX9 was detected in the CI group, but not in the other groups. 1: CI group. 2: Collagen group. 3: Sham group. 4: Chondrocytes (positive control). 5: No RNA (negative control).

To histologically validate the phenomenon of human cell-derived chondrogenesis at the repair sites, HLA-ABC staining for various lineages of human cells and hCOL2 for human cell-derived chondrocytes was performed using tissue samples obtained at 4 weeks after implantation. Transplanted human cells were detected as HLA-ABC-positive cells. Differentiated human chondrocytes derived from the transplanted cells were detected as hCOL2-positive cells at the chondral repair site in the CI group, while hCOL2-positive cells were not identified in the other groups (Figure 2A). The number of double-stained cells was higher in the CI group compared to the other groups (CI group 820 ± 52.3/mm^2^; Collagen group 0.0 ± 0.0/mm^2^; Sham group0.0 ± 0.0/mm^2^, respectively (*P* < 0.01 for CI group versus the other groups)).

RT-PCR analysis of tissue RNA, isolated from the transplantation sites, for human-specific chondrogenic markers (hCOL2 and hSOX9) revealed that expression of hCOL2 and hSOX9 was detected in the CI group but not in the other groups (Figure 2B).

### Macroscopic and histological recovery of regenerated cartilage

**Figure 3.**
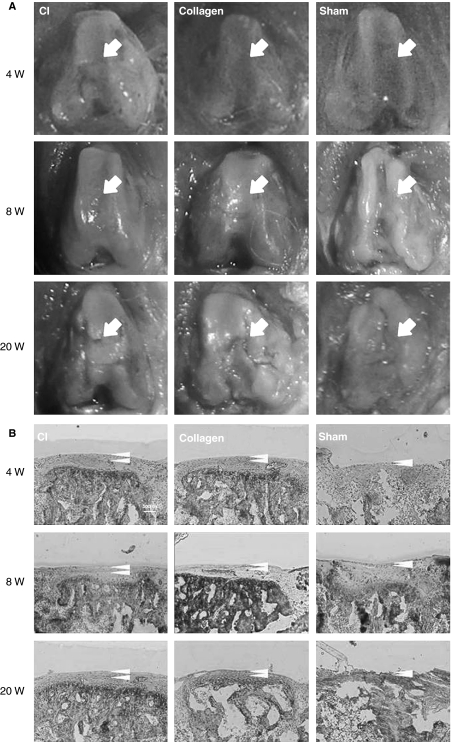
Macroscopic and histological evaluation at weeks 4, 8, and 20. A: Macroscopic assessment showing a better recovery as judged by a relatively smooth surface of the articular cartilage in the CI group compared to the other groups at weeks 4 and 8. In contrast, there was no apparent difference in the thickness of the articular cartilage between the three groups at week 20. Arrows indicate the defect area. B:Histological assessment with toluidine blue staining showing that the thickness of the articular cartilage was higher in the CI group than in the other groups at weeks 4, 8, and 20. However, at week 20, the thickness of the articular cartilage was decreasing in all groups. Arrow heads indicate the thickness of the articular cartilage.

Macroscopic assessment showed better recovery as judged by smooth surface of articular cartilage in the CI group when compared to the other groups at week 4 and 8 (Figure 3A). Histological assessment with toluidine blue staining (Figure 3B) showed that the thickness of the articular cartilage was significantly higher in the CI group than in the other groups at week 4 (CI group 15.3 ± 4.1 mm; Collagen group 6.0 ± 1.0 mm; Sham group 5.0 ± 2.0 mm, respectively; *P* < 0.05 for CI group versus the other groups). The differences between the CI group and the other groups also attained statistical significance at week 8 (CI group 27.5 ± 3.6 mm; Collagen group 9.7 ± 2.5 mm; Sham group 8.3 ± 1.5 mm, respectively;*P* < 0.05 for CI group versus the other groups). Semi-quantitative histological scorings at week 4 and 8 were better in the CI group than in the other groups (week 4: CI group 5.2 ± 1.1, Collagen group 9.0 ± 2.1, Sham group 15.6 ± 1.1; week 8: CI group 1 ± 0, Collagen group 7.8 ± 1.6, Sham group 14 ± 2.2, respectively;*P* < 0.05 for CI group versus the other groups).

However, at week 20, the surfaces of the articular cartilage did not differ among the three groups (Figure 3A). Histological assessment with toluidine blue staining at week 20 nevertheless showed that the articular cartilage was thicker in the CI group than in the other groups, although the decreasing trend of the thickness was maintained (CI group 13.0 ± 3.6 mm; Collagen group 2.6 ± 1.2 mm; Sham group 3.0 ± 1.0 mm, respectively; *P* < 0.05 for CI versus the other groups) (Figure 3B). In line with this, the histological score at week 20 was still better in the CI group than in the other groups (CI group 8 ± 2.8; Collagen group 12 ± 2.8; Sham group 22 ± 2.8, respectively;*P* < 0.05 for CI versus the other groups).

## Discussion

OA is characterized by the progressive loss of articular cartilage that leads to chronic pain and functional restrictions in affected joints. In the past decades, various surgical procedures including microfracture (16,17), autologous grafting procedures (18,19) and mosaicplasty (20,21) have been developed for the treatment of cartilage defects. However, there are no treatments that can reproducibly restore a normal articular surface. In recent years, chondrocyte implantation techniques, namely ACI, have emerged as a potential therapeutic option. Some studies also suggest a certain durability of the ACI, even in long-term follow-up studies, possibly because of its ability to produce hyaline-like cartilage and better integration with the adjacent articular surface (22,23).

Until now, the efficacy of ACI in patients suffering from OA has remained unclear. Several researchers reported the application of ACI to patients older than 45 years or who had failed prior treatments for articular cartilage defects of the knee (24,25). However, in these studies, OA-like changes of the articular cartilage was part of the excluding criteria. In the present study, we used collagen matrix, CaReS, as a third-generation autologous chondrocyte implant. The CaReS is a 3D collagen type 1 gel, and the thickness of the transplants can be adjusted to the thickness of the cartilage in each patient. Some clinical reports using the CaReS are encouraging, even in patella-femoral defects (26). However, there are no reports showing the usefulness of CaReS for cartilage repair in patients suffering from OA.

In this study, we used human chondrocytes in athymic nude rats. Using this xenotransplantation animal model, to confirm cartilage regeneration and maturation of human chondrocytes, molecular and immunohistological assessments using human-specific markers were conducted. RT-PCR and immunohistochemistry demonstrated survival of implanted chondrocytes at the defect site of the rat knee. This scientific approach provides us with useful information demonstrating the mechanistic effect of the third-generation ACI for OA. As the next step, we are planning to conduct a clinical trial to investigate the proof of concept of CaReS for use in focal OA patients. In addition, whereas a comparison of the CaReS technique with standard ACI is beyond the scope of the current study, comparative experiments to examine the effects of different ACI techniques in OA animal models are warranted. In the present study, we used human chondrocytes isolated from a relatively young volunteer. Although we intend to use this system for relatively young OA patients with focal OA regions, implantation of chondrocytes from older patients should also be investigated for the confirmation of the efficacy of the system also in that group of OA patients.

There was increased thickness of the articular cartilage and little deformity around the defect site when comparing the CI group with the other groups. This may be taken to indicate that the CI contributes to the stability of the cartilage. Up to 8 weeks, the thickness of articular cartilage at the defect sites in the CI group improved even when OA was getting worse due to the instability of the knee joint following ACL resection. However, at week 20, there was no increase of the thickness of the articular cartilage in the CI group. In the clinical setting, ligamentous instability is usually included in the excluding criteria of the study design. Hollander et al. described that OA did not inhibit tissue regeneration and might even have enhanced it, suggesting that degenerating tissues are primed for repair and require only the appropriate cellular cues and environment for proper regeneration of healthy tissue (27). Cartilage degradation in OA is considered to involve a delicate imbalance between anabolic and catabolic processes (28). Injured joints without OA may lack the endogenous anabolic pathways. Therefore, they must rely entirely on the introduced cells to produce a repair. In other words, joints with OA may repair relatively more easily than without OA.

There are some limitations in this study, such as for instance that we used bovine serum to prepare the graft. The use of human serum should preferably be investigated for clinical settings. In addition, we used a 1.5-mm diameter micro-drill in order to stabilize the implant and ensure that the medullary canal space was not reached. However, there might be a possibility that there might be an effect of chondroprogenitor cells on the articular cartilage. Hayes et al. described that the unique distribution of sulfation motifs within the microenvironment of superficial zone chondrocytes seems to designate early stages of stem/progenitor cell differentiation and is consistent with the fact that these molecules play a functional role in regulating aspects of chondrogenesis (29).

In the present study using a rat OA model, we showed the therapeutic potential of third-generation ACI for treating cartilage defects of knee OA. We believe our findings provide new insights into the treatment of OA and broaden the application of third-generation ACI for knee OA as a new surgical modality.
